# Supplementary material for: Identification of photoperiod-induced specific miRNAs in the adrenal glands of Sunite sheep (Ovis aries)
Source: Front Vet Sci. 2022 Jul 22;9:888207. doi: 10.3389/fvets.2022.888207 (PMC9354845; doi:10.3389/fvets.2022.888207)
Supplement: Supplementary file 1 [file Data_Sheet_1.ZIP › Supplementary file/miRDeep2 file.zip!/miRDeep2 file/Know_Structure/LP42A1/oar-mir-30b.pdf]

miRBase precursor : oar-mir-30b
Total read count : 35530
oar-miR-30b read count : 35219
remaining reads : 311

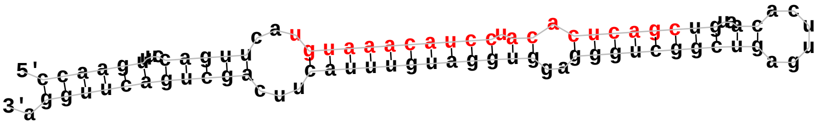

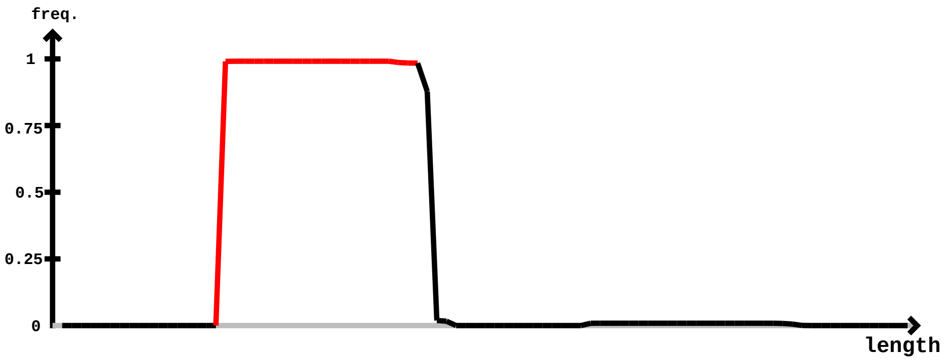

oar-miR-30b

```
5'- ccaaguuuccaguucauguaaacauccuacacucagcuguaacacuugagucggcugggagguggauguuuacuucagcugacuugga   -3'    exp
    (((((...(((((...((((((((((.((.((((((((..((......))))))))))..))))))))))))...)))))))))))).   reads    mm    sample
    ...............auguaaacauccuacacucagcu..................................................   3        0     seq
    ................uguaaacauccuacacu.......................................................   2        0     seq
    ................uguaaacaCccuacacuc......................................................   1        1     seq
    ................uguaaacauccuacacuc......................................................   127      0     seq
    ................uguaaacauccuacacuU......................................................   38       1     seq
    ................uguaaacauccuacacuca.....................................................   70       0     seq
    ................uguaaacauccuacacucag....................................................   16       0     seq
    ................uguaaacauccuacacucagc...................................................   3763     0     seq
    ................uguaaacauccuacacucagcu..................................................   30506    0     seq
    ................uguaaacauccuacacucagcug.................................................   93       0     seq
    ................uguaaacauccuacacucagcugu................................................   576      0     seq
    ................uguaaacauccuacacucagcugua...............................................   5        0     seq
    .................guaaacauccuacacucagcu..................................................   19       0     seq
    ......................................................cugggagguggauguuuacu..............   28       0     seq
    ......................................................cugggagguggauguuuacuu.............   81       0     seq
    ......................................................cugggagguggauguuuacuuc............   180      0     seq
    ......................................................cugggagguggauguuuacuuca...........   15       0     seq
    .......................................................ugggagguggauguuua................   1        0     seq
    .......................................................ugggagguggauguuuacuu.............   1        0     seq
    .......................................................ugggagguggauguuuacuuc............   2        0     seq
    .......................................................ugggagguggauguuuacuuca...........   3        0     seq
```
